# Supplementary material for: Content-rich biological network constructed by mining PubMed abstracts
Source: BMC Bioinformatics. 2004 Oct 8;5:147. doi: 10.1186/1471-2105-5-147 (PMC528731; doi:10.1186/1471-2105-5-147)
Supplement: Additional File 2 — The original results of the above study (non-essential files are deleted to keep the file size under the limit set by BMC bioinformatics). [file 1471-2105-5-147-S2.bz2 › chilibotAdditionalFile2/dip20/273ID1922387E1093/html/MAPK1_JUN.html]

 


 **MAPK1** and **JUN** 
  
Found 2098 abstracts in PubMed, retrieved 20.  
 

 What does Google say? 
 PDF only 
| .edu only 

---

**Interactive relationship** (e.g. stimulation, inhibition, etc)

**Inhibitory relationship**- SP600125, an inhibitor of c  **jun**  N terminal kinase, activates CREB by a p38 MAPK  [ **MAPK1** ]  mediated pathway.  Ref: 12878189 Biochem Biophys Res CommunBiochem Biophys Res Commun,
**Stimulatory relationship**- This selective MAPK  [ **MAPK1** ]  inhibition was associated with a reduction in AP 1  [ **JUN** ]  activity, with no affect on NF kappaB activity.  Ref: 12865664 Shock, 2003
**Neutral relationship**- Apoptosis signal regulating kinase 1 ASK1, a MAPK  [ **MAPK1** ]  kinase kinase MAPKKK protein, is known to activate both c  **Jun**  N terminal kinase JNK and p38 via MAPK  [ **MAPK1** ]  kinase MKK 4 7 and MKK3 6, respectively.  Ref: 12869527 J Biochem (Tokyo), 2003

**Non-interactive relationship** (e.g. studied together, co-existance, homology, etc.)

- Sorbitol resulted in activation of the extracellularly responsive kinase ERK  [ **MAPK1** ] , p54 c  **Jun**  N terminal kinase JNK and protein kinase B PKB.  Ref: 12881532 J Biol ChemJ Biol Chem, 2003
- Apoptosis signal regulating kinase1 ASK1 is a ubiquitously expressed mitogen activated protein kinase kinase kinase MAPKKK that activates the SEK1 c  **Jun**  N terminal kinase JNK and MKK3 MKK6 p38 MAPK  [ **MAPK1** ]  signaling cascades.  Ref: 12878192 Biochem Biophys Res Commun, 2003
- 2 We examined the role of the mitogen activated protein kinase MAPK  [ **MAPK1** ]  c  **jun**  N terminal kinase JNK in TNFalpha and IL 1beta induced GM CSF, RANTES and IL 8 production in HASMC by using a novel specific inhibitor for JNK SP600125.  Ref: 12871843 Br J Pharmacol, 2003
- We identified that serum starvation and Dox reduced phosphorylation of mitogen activated protein kinase extracellular signal regulated kinase ERK  [ **MAPK1** ]  kinase MEK, protein kinase C PKC alpha beta and c  **Jun**  NH 2 terminal kinase.  Ref: 12883037 Mol Cancer Ther, 2003
- The recovery of aconitase activity also correlates with the activation of members of the MAPKs, p38, c  **Jun**  N terminal kinase JNK and ERK  [ **MAPK1** ] , and the activation p38 and JNK is attenuated by inhibition of guanylate cyclase.  Ref: 12865320 Endocrinology, 2003
- Such molecules include nuclear transcription factors e.g. GATA 4, c  **Jun** , protein kinases e.g. 90 kDa ribosomal protein S6 kinase, MAPK  [ **MAPK1** ]  activated protein kinase 2, and ion exchangers channels e.g. the Na H exchanger 1.  Ref: 12878473 J Mol Cell Cardiol, 2003
- While overexpression of wild type Ras enhanced CpG ODN induced extracellular signal regulated kinase ERK  [ **MAPK1** ] , c  **Jun**  NH 2 terminal kinase JNK and NF kappaB activation, overexpression of RasN17 inhibited CpG ODN induced ERK  [ **MAPK1** ] , JNK and NF kappaB activation.  Ref: 12867418 J Biol ChemJ Biol Chem, 2003
- In this study, we investigated the role of c  **Jun**  NH2 terminal kinase JNK, a member of the mitogen activated protein kinase MAPK  [ **MAPK1** ]  family, in lipopolysaccharide LPS stimulated inducible nitric oxide synthase iNOS expression and nitric oxide NO production in J774 murine macrophages.  Ref: 12869635 Mol PharmacolMol Pharmacol, 2003
- These results suggest that induction of the MMP 13 gene by TNF alpha is mediated by ERK  [ **MAPK1** ] , p38, and JNK MAP kinases as well as AP 1  [ **JUN** ]  and NF kappaB transcription factors.  Ref: 12878172 Exp Cell Res, 2003
